# Automated measurement of spinopelvic alignment parameters using a spine planning software: a validation study

**DOI:** 10.1007/s43390-025-01216-7

**Published:** 2025-11-27

**Authors:** Ahmad Almahozi, Anton Früh, Tarik Alp Sargut, Tizian Rosenstock, Claudius Jelgersma, Anna L. Roethe, Dimitri Tkatschenko, Peter Truckenmueller, Joan Alsolivany, Kiarash Ferdowssian, Nils Hecht, Peter Vajkoczy, Lars Wessels

**Affiliations:** Department of Neurosurgery, Charité – Universitätsmedizin Berlin, Corporate member of Freie Universität Berlin, Humboldt-Universität Zu Berlin, and Berlin Institute of Health, Berlin, Germany

**Keywords:** Spinopelvic alignment, Automated measurement, Brainlab Elements, Spine planning software, Validation study, Spinal deformity

## Abstract

**Background:**

Accurate assessment of spinopelvic alignment is essential for managing adult spinal deformities. This study validates the Brainlab Elements Spine Planning software for automated measurement of spinopelvic parameters, comparing it with manual methods.

**Methods:**

Spinopelvic parameters were measured manually and with the software in 21 patients with degenerative spinal disease, including instrumented and non-instrumented spines. Accuracy, intraobserver, and interobserver reliability were evaluated using Bland–Altman plots and intraclass correlation coefficients (ICCs). Measurement times were also compared.

**Results:**

The software showed high reliability (ICC = 1), while manual measurements ranged from fair to excellent reliability (ICC 0.44–0.99). Bland–Altman plots indicated strong agreement between automated and manual measurements, though variability was noted for certain parameters. Automated measurements were significantly faster, averaging 62 s versus 227 s in manual measurements (p < 0.001), though 76.2% of cases had at least one parameter that could not be measured automatically, most frequently the sagittal vertical axis (SVA) and several coronal parameters.

**Conclusion:**

The Brainlab Elements software provides accurate, reproducible, and time-efficient spinopelvic measurements for parameters it successfully captures. However, frequent failures in assessing SVA and coronal plane parameters automatically suggest that further refinement of the software is necessary.

## Introduction

The analysis of sagittal alignment is essential for the diagnosis and management of adult spinal deformities. Several studies have demonstrated significant correlations between radiographic spinopelvic parameters and clinical outcomes, including pain, disability, and health-related quality of life [1, 2]. A precise preoperative assessment of these parameters is critical for accurately determining the required degree of spinal correction, as inadequate assessment can lead to suboptimal postoperative alignment and poor clinical outcomes [3].

Various dedicated measurement software solutions have been reported to offer greater accuracy and reliability compared to traditional manual measurement techniques [4–7]. Nevertheless, the clinical adoption of such software has remained limited, as many require multiple additional steps such as downloading and transferring DICOM files, manual identification of numerous anatomical landmarks, or image pre-processing. These factors increase user dependency, prolong measurement time, and reduce practicality in daily clinical workflows [7, 8].

The Brainlab Elements Spine Planning software (Brainlab, Munich, Germany) automatically proposes labeling of vertebral bodies and generates a set of spinopelvic parameters based on detected anatomical landmarks. Users may manually edit and adjust these labels in cases involving altered spinal anatomy due to deformities or neoplasia. The software provides immediate access to patient datasets from any workstation connected to the Brainlab server, allowing direct interaction with images in the Digital Imaging and Communications in Medicine (DICOM) format.

The objective of this study was to evaluate the accuracy and reliability of the Brainlab Elements Spine Planning software in measuring selected spinopelvic parameters. Measurements generated by the Brainlab software were compared to traditional manual measurements obtained using Picture Archiving and Communication Systems (PACS) tools.

## Materials and methods

### Patient population

**Fig. 1:**
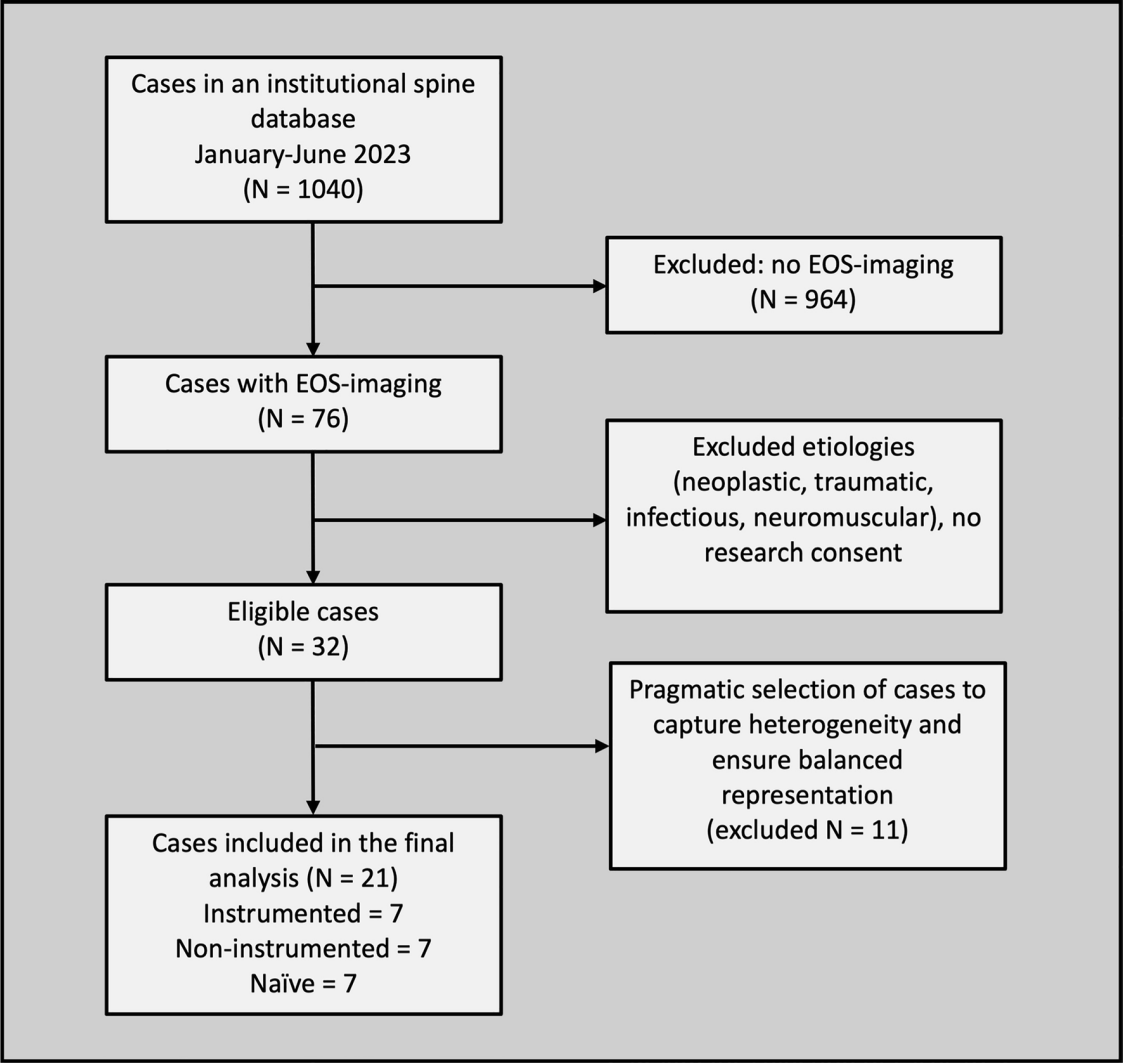
CONSORT flow diagram of patient selection

Local ethics committee approval was obtained prior to study initiation. Subjects were retrospectively selected from a spinal database at a single institution. Inclusion criteria were: age greater than 18 years and availability of low-dose, full-body (head-to-feet) biplanar stereoradiographic images (EOS imaging, Paris, France) acquired between January and June 2023. Patients were excluded if their spinal deformity resulted from neoplastic, neuromuscular, infectious, or traumatic etiologies. To represent clinical variability, 21 subjects – including naïve, instrumented, and non-instrumented cases – were selected by a senior spine surgeon according to the predefined inclusion and exclusion criteria. The final sample size was determined pragmatically from the available EOS database during the study period, with the aim of capturing the heterogeneity of routine clinical practice. The process of patient identification, exclusions, and final cohort selection is summarized in the CONSORT flow diagram (Fig. 1).

### Image protocol

All patients underwent low-dose, full-body (head-to-feet) biplanar stereoradiographic imaging using the EOS imaging system. Images were acquired in a standardized, weight-bearing, free-standing position with both femoral heads clearly visible. Patients stood with hips and knees fully extended, elbows flexed, and hands placed on the contralateral clavicles to prevent superimposition with spinal structures [9]. The obtained images were initially uploaded to a PACS in DICOM format and subsequently transferred for further measurement analysis using the designated spinal planning software.

### Sagittal spinopelvic parameters

**Fig. 2:**
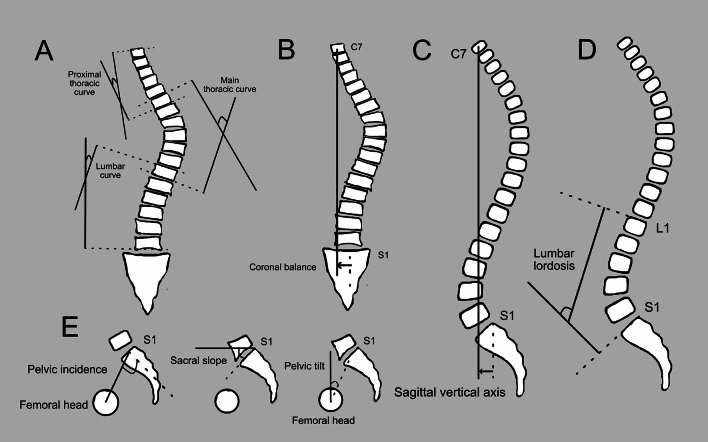
Spinopelvic and coronal parameters measured. (**A**) proximal thoracic curve, main thoracic curve, and lumbar curve; (**B**) coronal balance; (**C**) sagittal vertical axis; (**D**) lumbar lordosis; (**E**) pelvic incidence, sacral slope, and pelvic tilt

The spinopelvic parameters assessed included pelvic tilt (PT), pelvic incidence (PI), sacral slope (SS), lumbar lordosis (LL), sagittal vertical axis (SVA), coronal balance (CB), main thoracic curve (MTC), proximal thoracic curve (PTC), and lumbar curve (LC), as illustrated in Fig. 2. For the measurement of MTC, PTC, and LC, the apex vertebra (defined as the vertebra showing the greatest deviation from the spinal midline) and the corresponding end vertebrae (defined as those with maximal tilt toward the curve apex) were identified. The curve with the largest Cobb angle was classified as the major curve, while all remaining curves were considered minor [10, 11].

### Measurement process

**Fig. 3:**
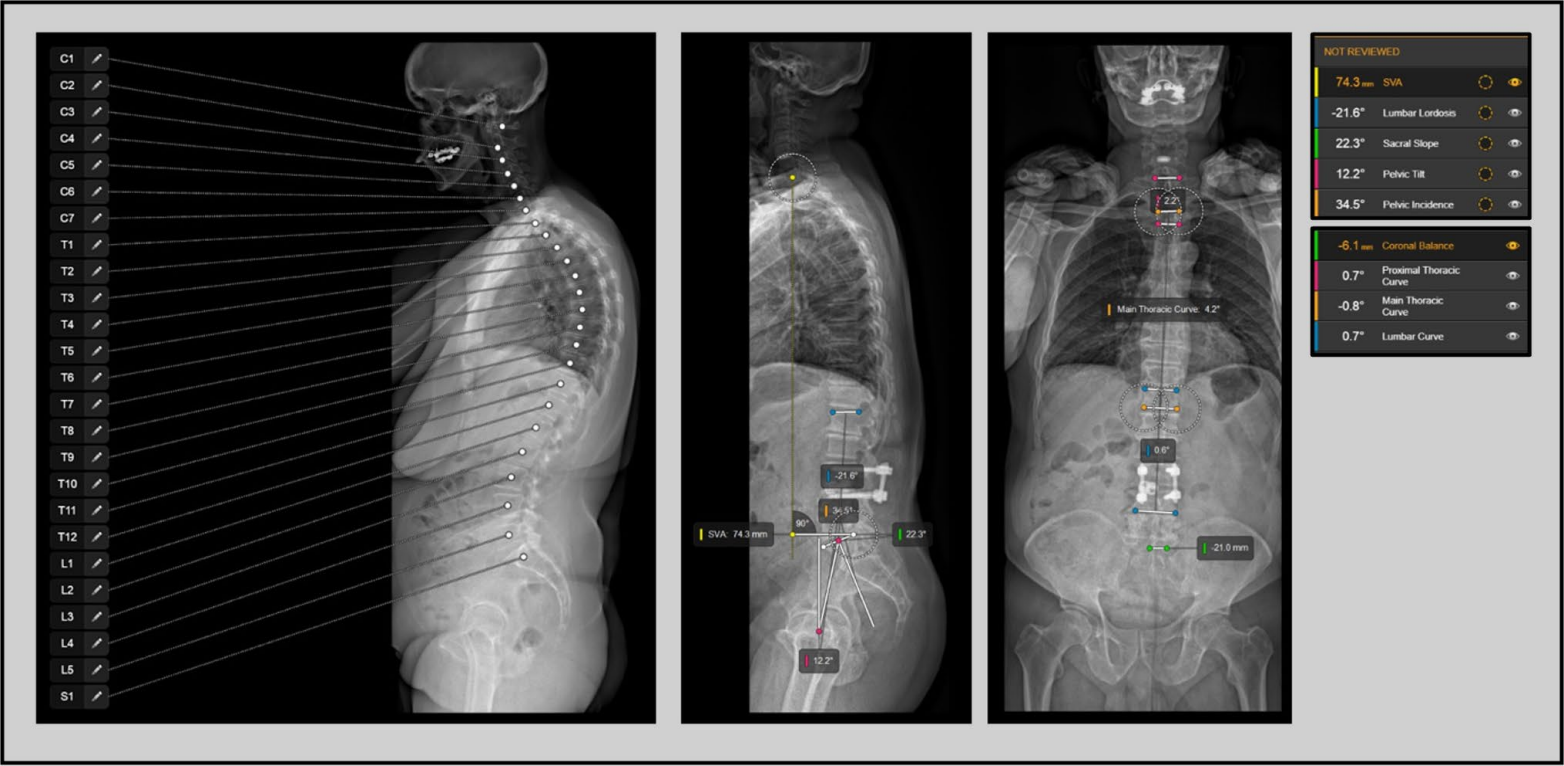
Representative scaled sagittal and coronal radiographs showing the vertebral bodies as identified by the software and the spinopelvic parameters derived

**Fig. 4:**
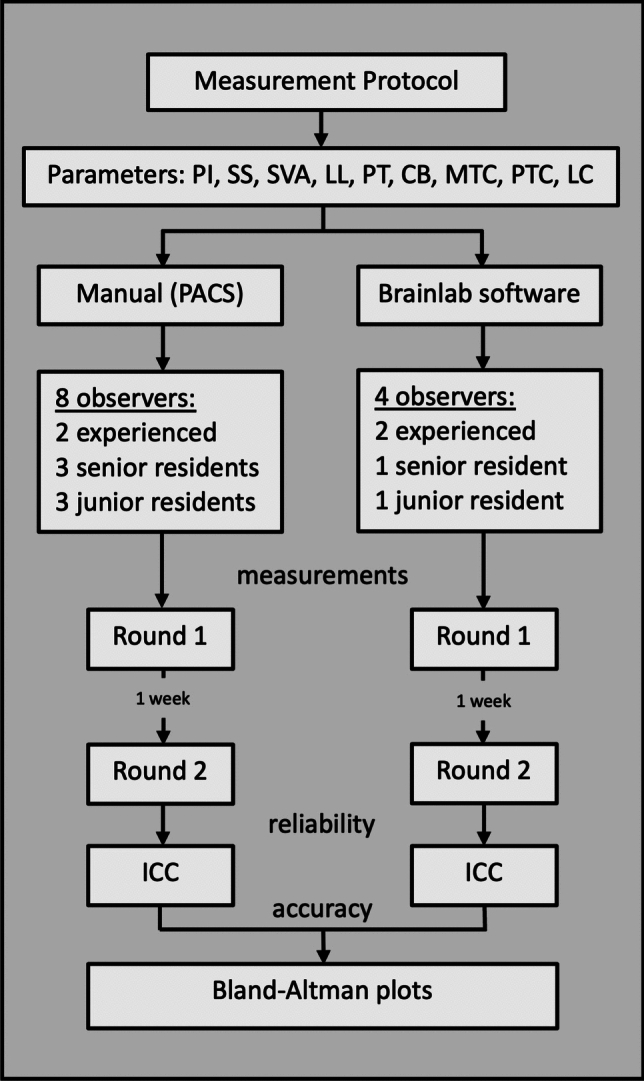
Measurement protocol. *PI* Pelvic incidence; *SS* Sacral slope; *LL* Lumbar lordosis; *PT* Pelvic tilt; *SVA* Sagittal vertical axis; *CB* Coronal balance; *MTC* Main thoracic curve; *PTC* Proximal thoracic curve; *LC*: Lumbar curve; *ICC* Intraclass correlation coefficient

The Brainlab Elements Spine Planning software was employed for automated measurements. After selecting the EOS images, the software automatically identified and labeled vertebral bodies. Users were then able to confirm or manually adjust the suggested labels. Once confirmed, the software immediately generated a complete set of spinopelvic parameters (Fig. 3). For manual measurements, basic line and angle tools available within a PACS interface (MERLIN, Phoenix-PACS, Freiburg, Germany) were used. Manual measurements were conducted twice per patient by eight observers (two experienced spine surgeons, three senior neurosurgical residents, and three junior neurosurgical residents), with a one-week interval between measurement rounds. Observers were blinded to their initial measurements during the second round. An additional four observers (two spine surgeons and two neurosurgical residents – one senior and one junior) performed measurements using the software (Fig. 4).

### Statistical analysis

The validation of the Brainlab Elements Spine Planning software involved two primary aspects: the assessment of accuracy and evaluation of reliability. To determine accuracy, mean values, standard deviations (SD), and mean absolute differences for each parameter were calculated, comparing measurements obtained with the software against those obtained manually. Agreement between these two measurement methods was assessed through Bland–Altman analysis [12].

Reliability was assessed by calculating intraclass correlation coefficients (ICCs) for each parameter. ICC values were calculated to evaluate intraobserver reliability (consistency of repeated measurements by the same observer across two rounds) and interobserver reliability (agreement among different observers within each measurement round). ICC results were interpreted according to the following thresholds: 0.90–1.0 (excellent), 0.70–0.89 (good), 0.50–0.69 (fair), 0.25–0.49 (low), and 0.24 or lower (poor) [13].

Measurement durations for each case were recorded for both the manual and automated method. Paired t-tests were conducted separately for each measurement round to compare the time required by each method. Additionally, a 2 × 2 within-subject repeated-measures ANOVA was conducted on measurement time with factors Method (automated vs. manual) and Round (1 vs. 2), including the Method × Round interaction; significance was set at α = 0.05 and effect sizes are reported as partial η^2^. Statistical analyses were performed using SPSS Version 20.0 (IBM, Armonk, USA) and Excel Version 16.80 (Microsoft, Redmond, USA).

## Results

### Accuracy

The Brainlab Elements Spine Planning software failed to measure the SVA in 5 patients (23.8%). Additionally, the software was unable to identify several coronal parameters: CB in 8 patients (38.1%), PTC in 11 patients (52.4%), MTC in 5 patients (23.8%), and LC in 2 patients (9.5%). Moreover, the software incorrectly recognized coronal images as sagittal in two cases, resulting in incorrect calculations of sagittal parameters rather than the intended coronal parameters. In total, 16 patients (76.2%) had at least one parameter that the software failed to generate. Across all patients and parameters, 39 of 189 expected measurements (20.6%) could not be obtained, corresponding to an overall success rate of 79.4%. Among these cases, seven involved instrumented spines and nine had non-instrumented spines. To ensure consistency, corresponding manual measurements for patients with incomplete automated measurements were excluded from subsequent analysis.

Successfully measured spinopelvic parameters, including mean values, mean absolute differences, minimum and maximum absolute differences, and standard deviations, are summarized in Tables 1 and 2.

**Table 1:** Mean values and standard deviations of spinopelvic parameters measured manually and automatically on both rounds. Automated measurements were identical on both rounds

	Manual		Automated	
	Round 1	Round 2	Round 1	Round 2
	Mean ± SD	Mean ± SD	Mean ± SD	Mean ± SD
PI (°)	59.3 ± 16.1	58.8 ± 15.3	58.4 ± 15.7	58.4 ± 15.7
SS (°)	35.5 ± 11.1	36.5 ± 11.1	36.2 ± 11.6	36.2 ± 11.6
SVA (mm)	41.9 ± 49	42.4 ± 48.6	39.9 ± 49	39.9 ± 49
LL (°)	−32.8 ± 30.4	32.6 ± 30.3	−36.7 ± 31.7	−36.7 ± 31.7
PT (°)	22.8 ± 9.6	22.3 ± 9.6	22.2 ± 11.6	22.2 ± 11.6
CB (mm)	−3.6 ± 21.4	−3.4 ± 21.4	−3.35 ± 24.9	−3.35 ± 24.9
PTC (°)	3.3 ± 4.6	3.8 ± 3.9	1.9 ± 6.3	1.9 ± 6.3
MTC (°)	−0.2 ± 5.2	−0.1 ± 5.2	−0.35 ± 5.4	−0.35 ± 5.4
LC (°)	1.3 ± 7.1	1.4 ± 6.9	0.74 ± 6.4	0.74 ± 6.4

**Table 2:** Differences between manual and Brainlab measurements

	Mean absolute difference	Mean absolute difference (SD)	Minimum absolute difference	Maximum absolute difference
PI (°)	1.9	0.9	0.4	3.7
SS (°)	2.8	2.5	0.3	9.6
SVA (mm)	3.9	3.9	0.1	13.4
LL (°)	6.3	3.8	0.1	17.0
PT (°)	2.4	2.8	0.3	11.7
CB (mm)	6.1	4.9	1.8	19.9
PTC (°)	2.2	2.9	0.1	8.7
MTC (°)	2.2	1.6	0.02	5.0
LC (°)	1.6	1.0	0.05	3.6

The largest mean absolute differences between manual and automated measurements were found for the SVA (3.9 mm ± 3.9 mm), LL (6.3° ± 3.8°), and CB (6.1 mm ± 4.9 mm). Excluding these parameters, all other measurements demonstrated differences within 3°. The smallest absolute differences observed were consistently below 0.5° for all angular parameters. Coronal parameters (PTC, MTC, and LC), excluding CB, generally showed the smallest absolute differences between automated and manual measurements (Tables 1, 2).

**Fig. 5:**
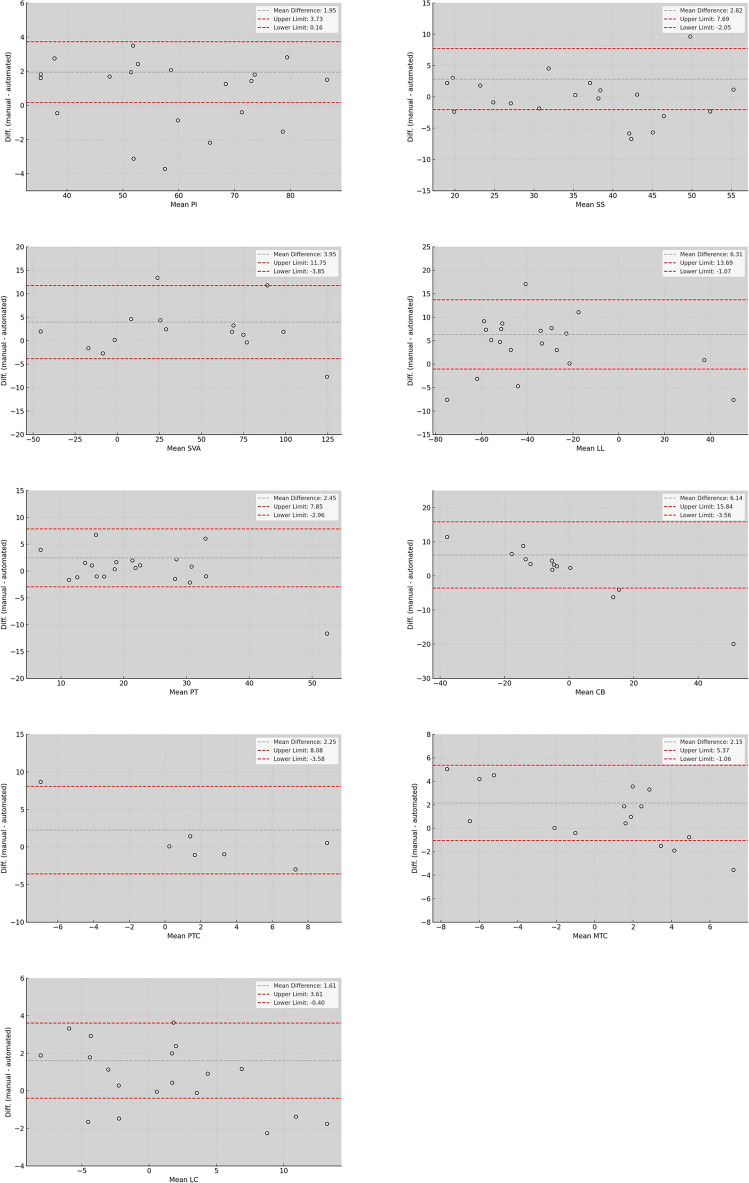
Bland–Altman plots showing the differences between manual and automated measurements for pelvic incidence (PI), sacral slope (SS), sagittal vertical axis (SVA), lumbar lordosis (LL), pelvic tilt (PT), coronal balance (CB), proximal thoracic curve (PTC), main thoracic curve (MTC), and lumbar curve (LC)

Bland–Altman plots demonstrate general consistency between manual and automated measurements. However, the agreement varied among parameters. Specifically, LC (mean difference 1.61, limits of agreement −0.40 to 3.41), PI (mean difference 1.95, limits of agreement 0.16 to 3.73), and MTC (mean difference 2.15, limits of agreement −1.06 to 5.17) displayed the highest agreement with minimal bias and narrow limits of agreement. Conversely, SVA (mean difference 3.95, limits of agreement −3.15 to 11.75), CB (mean difference 6.14, limits of agreement −3.56 to 15.84), and LL (mean difference 6.31, limits of agreement −1.07 to 13.69) showed lower agreement, characterized by higher biases and broader limits of agreement (Fig. 5).

### Reliability

Intraobserver and interobserver intraclass correlation coefficients (ICCs) are summarized in Tables 3 and 4. For manual measurements, ICC values ranged between 0.44 and 0.99, indicating fair to excellent reliability. The lowest mean intraobserver ICC was observed for CB (0.85), whereas the lowest mean interobserver ICC values were observed for PTC (0.53) and MTC (0.67). Experienced observers (observers 1–2) demonstrated greater reliability, with mean intraobserver and interobserver ICCs of 0.96 and 0.90, respectively, compared to less experienced observers with lower ICC values (mean intraobserver ICC: 0.92; mean interobserver ICC: 0.77).

**Table 3:** Intraobserver correlation between measurements obtained on both rounds by eight observers manually and four using Brainlab software

	ICC (manual)									ICC (automated)				
	1*	2*	3	4	5	6	7	8	Mean	1*	2*	3	4	Mean
PI	0.92	0.96	0.97	0.95	0.99	0.99	0.81	0.88	**0.93**	1	1	1	1	**1**
SS	0.99	0.98	0.92	0.96	0.99	0.99	0.82	0.92	**0.95**	1	1	1	1	**1**
SVA	0.99	0.99	0.99	0.99	0.92	0.98	0.87	0.82	**0.94**	1	1	1	1	**1**
LL	0.93	0.99	0.99	0.81	0.89	0.91	0.92	0.91	**0.92**	1	1	1	1	**1**
PT	0.89	0.89	0.99	0.89	0.98	0.88	0.87	0.74	**0.89**	1	1	1	1	**1**
CB	0.99	0.99	0.98	0.65	0.96	0.94	0.87	0.44	**0.85**	1	1	1	1	**1**
PTC	0.94	0.99	0.85	0.96	0.94	0.99	0.93	0.95	**0.94**	1	1	1	1	**1**
MTC	0.95	0.99	0.90	0.99	0.98	0.97	0.98	0.99	**0.97**	1	1	1	1	**1**
LC	0.97	0.97	0.98	0.98	0.99	0.99	0.99	0.99	**0.98**	1	1	1	1	**1**

^*^Experienced observers

**Table 4:** Interobserver correlation between measurements obtained by the observers on both rounds. ICC means are calculated for all observers

	ICC (manual)							ICC (automated)**		
	Round 1			Round 2				Round 1	Round 2	
	1–2*	3–8	1–8	1–2*	3–8	1–8	Mean	1–4	1–4	Mean
PI	0.99	0.78	0.94	0.99	0.72	0.74	**0.84**	1	1	**1**
SS	0.99	0.84	0.78	0.88	0.82	0.72	**0.75**	1	1	**1**
SVA	0.99	0.98	0.98	0.96	0.95	0.95	**0.97**	1	1	**1**
LL	0.98	0.92	0.93	0.99	0.92	0.92	**0.93**	1	1	**1**
PT	0.99	0.78	0.82	0.79	0.77	0.92	**0.87**	1	1	**1**
CB	0.88	0.96	0.92	0.96	0.65	0.73	**0.86**	1	1	**1**
PTC	0.91	0.51	0.60	0.89	0.32	0.47	**0.53**	1	1	**1**
MTC	0.65	0.65	0.66	0.59	0.67	0.67	**0.67**	1	1	**1**
LC	0.93	0.84	0.84	0.92	0.85	0.83	**0.84**	1	1	**1**

^*^Experienced observers

^**^Results are not stratified based on experience as ICC was identical across all observers

Automated measurements, however, yielded identical values for all parameters across all observers and measurement rounds, with no manual adjustments required during automated labeling in this cohort, resulting in ICCs of 1 and indicating excellent interobserver and intraobserver reliability and repeatability.

### Measurement times

The mean time required to perform a complete spinopelvic measurement analysis during round 1 was significantly shorter with the software (62 ± 27 s) compared to manual measurements (227 ± 72 s), representing a 3.6-fold reduction (t(20) = −9.952, p < 0.001). Similarly, during round 2, measurement times remained significantly shorter with the automated method (58 ± 21 s) compared to the manual method (221 ± 65 s; t(20) =  −10.010, p < 0.001).

On a 2 × 2 repeated-measures ANOVA (within-subjects factors: Method and Round), measurement time showed a large main effect of Method (F(1,20) = 105.74, p < 0.001), with automated measurements significantly faster than manual measurements. There was also a main effect of Round (F(1,20) = 25.97, p = 0.0001), reflecting shorter times in round 2, and a small Method × Round interaction (F(1,20) = 6.28, p = 0.0209), indicating a slightly smaller manual–automated difference in round 2 (144.5 s) than in round 1 (163.9 s). Within-round paired comparisons remained significant (round 1: t(20) =  − 9.95, p < 0.001; round 2: t(20) =  − 10.01, p < 0.001).

## Discussion

Measuring spinopelvic parameters can be challenging and time-consuming. Several semi-automated computer-assisted measurement tools have recently emerged and have demonstrated faster performance, and in certain cases, superior reliability and reproducibility compared to traditional manual measurement methods [4–7]. Despite the clear advantages, many surgeons admit to not using them in daily practice, as some require extensive identification of anatomical landmarks or have difficult user interfaces [8].

In the current study, the average time required to measure all assessed spinopelvic parameters was significantly shorter using the Brainlab Elements Spine Planning software (62 s) compared to the traditional manual method (226 s), representing a 3.6-fold improvement. Although, the list of parameters assessed in this study was not as comprehensive as those reported by other studies, our findings regarding measurement speed align closely with the literature and demonstrate a comparable advantage [6, 7].

The rapid acquisition of measurements using the Brainlab software can be attributed to its fully automated vertebral identification and intuitive interface, requiring minimal user input. The user is only required to select the relevant images; the software then automatically identifies and labels vertebral bodies. Users can subsequently confirm or adjust the labeling, after which all parameters are instantly generated. In contrast, other software solutions necessitate sequential manual identification of multiple anatomical landmarks before generating spinopelvic parameters. For instance, Surgimap (Nemaris, Methuen, USA), a widely used semi-automated software, requires users to identify five anatomical landmarks (femoral head, upper endplate of S1, L1, T1, and lower endplate of C2) before automatic parameter calculation [4, 6]. Other traditional or semi-automated methods require identification of up to 12 landmarks, potentially increasing measurement time, operator dependency, and susceptibility to human error [14].

While previous validation studies have predominantly involved healthy populations [6], our study included patients with both untreated degenerative deformities and previously instrumented spines, providing a realistic clinical setting. Furthermore, we incorporated observers of varying expertise levels to assess consistency across different experience profiles. For manual measurements, reliability ranged from fair to excellent, with experienced observers demonstrating higher intraobserver and interobserver reliability than less-experienced observers. Notably, coronal plane parameters (CB, PTC, MTC) exhibited lower reliability compared to sagittal parameters, a finding consistent with previous studies reporting greater difficulty and variability in manually measuring thoracic curves on coronal imaging, with reported variabilities of 3°–10° [15–18]. Likewise, prior studies involving semi-automated measurement software have reported varied error and observer agreement. For instance, endplate and curve measurements have been found to be less reliable compared to point measurements [13], with reported variabilities ranging from 4° to 7° in some studies [7, 19, 20]. Measurement of lumbar lordosis (LL), in line with prior studies, remains challenging and less accurate due to potential superimposition by rib structures, hindering clear identification of lumbar vertebral endplates [21].

Despite these limitations, automated and semi-automated methods consistently show improved reliability compared to fully manual methods [4–6]. In this study, the Brainlab Elements Spine Planning software generated identical values across all measurement rounds, independent of observer background, and consistently outperformed manual measurements, which showed greater variability across different levels of experience. Although no formal subgroup analysis was performed, future studies could address more directly how observer experience influences relative performance. Additionally, measurement differences compared to manual methods were minimal (less than 3°), with the exception of the SVA at 2 mm difference, the LL at 3.9°, and the CB at 6.1 mm. Thus, while acknowledging specific limitations, the software demonstrated robust reproducibility and acceptable accuracy for parameters successfully measured.

This study specifically evaluated nine clinically relevant spinopelvic parameters, which, although representative of commonly used metrics, do not encompass all sagittal and coronal measurements available. Other parameters automatically generated by the software, such as C7-slope, T1-slope, thoracic kyphosis, and L5/S1 spondylolisthesis, were not validated herein. On the other hand, parameters such as T4–pelvic angle (T4PA) and L1–pelvic angle (L1PA), which are increasingly reported in the current spinal deformity literature, are not generated by the software and were therefore not included in this validation. Furthermore, our study did not explicitly evaluate transitional anatomy (e.g., sacralization of L5 or lumbarization of S1), and it remains unclear how the software performs in such cases. Manual verification of vertebral numbering is therefore essential, and future studies should directly assess software reliability in transitional variants. An additional limitation of this study was the indirect assessment of the software’s user-friendliness based solely on measurement duration rather than a formal usability analysis. Moreover, the relatively small sample size of 21 cases, determined pragmatically from the EOS database during the study period, also limits the generalizability of our findings. Although the cohort was designed to capture the heterogeneity of routine clinical practice by including naïve, instrumented, and non-instrumented cases, the results should be interpreted cautiously. Larger, randomly selected, multi-center cohorts will be required to confirm and extend these findings.

Finally, a notable limitation of this study is the high rate of measurement failure observed with the software. Sixteen patients (76.2%) had at least one parameter that could not be measured automatically, most frequently affecting SVA and coronal parameters (CB, PTC, MTC, LC). When considered across all patients and parameters, 39 of 189 expected measurements (20.6%) were not obtained, corresponding to an overall success rate of 79.4%. Published overall detection rates vary: for example, Löchel et al. recently reported that a deep learning–based algorithm achieved detection success in 91.5% of preoperative cases and 84.8% of postoperative cases [22]. The reasons for these failures remain unclear, as neither instrumentation nor image quality consistently explained detection success in our cohort. Given the small but heterogeneous sample, reliable extrapolation or stratification of failure patterns was not possible, and our findings should therefore be regarded as exploratory. Larger studies are required to stratify patients more robustly, assess modality-specific effects, and clarify the underlying causes of detection failures. Corresponding manual measurements for incomplete cases were excluded from comparative analysis, limiting validation of these parameters. Similar challenges have been reported with other AI-based and automated software, often related to anatomical variability, implant-associated artifacts, femoral head pathology (e.g., obscuration after hip arthroplasty), and landmark misidentification due to out-of-plane imaging artifacts [23, 24]. In addition, the software evaluated in this study was not claimed to be EOS-specific. While several deep-learning algorithms have shown robust validation results for lateral whole-spine EOS images [21, 25], expanding software training datasets to include diverse anatomical variations, imaging conditions, and modalities is necessary to enhance robustness, accuracy, and clinical utility. EOS was selected for this study as it represents the institutional standard of care and provides standardized, calibrated, low-dose, full-body, weight-bearing images with fewer magnification and stitching errors compared to conventional radiographs. Nevertheless, the exclusive use of EOS may have contributed to the high rate of automated measurement failure observed in this study, highlighting the need to evaluate the software across different imaging modalities. Future studies should therefore directly compare EOS with conventional long-standing radiographs to determine whether automated detection performance is modality dependent.

## Conclusion

In this validation study, the Brainlab Elements Spine Planning software demonstrated significantly faster measurements and high reproducibility for successfully assessed parameters. However, limitations were observed, with a substantial proportion of failed automated measurements (approximately 76% of patients had at least one unmeasured parameter). Particularly, sagittal vertical axis (SVA) and several coronal parameters frequently failed to be detected by the software. Thus, while the software shows promise, especially in efficiency and reproducibility for successfully measured parameters, significant improvements in parameter detection reliability and software robustness are necessary before recommending widespread clinical implementation.

## Data Availability

The datasets are available upon reasonable request.

## References

[1] Glassman SD, Berven S, Bridwell K, Horton W, Dimar JR (2005) Correlation of radiographic parameters and clinical symptoms in adult scoliosis. Spine 30:682–688. 10.1097/01.BRS.0000155425.04536.F7 15770185 10.1097/01.brs.0000155425.04536.f7

[2] Gao A, Wang Y, Yu M, Wei F, Jiang L, Liu Z, Liu X (2020) Association between radiographic spinopelvic parameters and health-related quality of life in de novo degenerative lumbar scoliosis and concomitant lumbar spinal stenosis. Spine 45:E1013–E1019. 10.1097/BRS.0000000000003471 32118697 10.1097/BRS.0000000000003471 PMC7386863

[3] Liu S, Moal B, Lafage V, Maier SP, Challier V, Skalli W, Protopsaltis TS, Errico TJ, Schwab FJ (2014) Discrepancies in preoperative planning and operative execution in the correction of sagittal spinal deformities. Spine J 14:S3–S4. 10.1016/j.spinee.2014.08.020

[4] Wu W, Liang J, Du Y, Tan X, Xiang X, Wang W, Ru N, Le J (2014) Reliability and reproducibility analysis of the Cobb angle and assessing sagittal plane by computer-assisted and manual measurement tools. BMC Musculoskelet Disord 15:33. 10.1186/1471-2474-15-33 24502397 10.1186/1471-2474-15-33 PMC3922010

[5] Dimar JR, Carreon LY, Labelle H, Djurasovic M, Weidenbaum M, Brown C, Roussouly P (2008) Intra- and inter-observer reliability of determining radiographic sagittal parameters of the spine and pelvis using a manual and a computer-assisted methods. Eur Spine J 17:1373–1379. 10.1007/S00586-008-0755-1 18726124 10.1007/s00586-008-0755-1 PMC2556466

[6] Fleiderman Valenzuela JG, Cirillo Totera JI, Turkieltaub DH, Echaurren CV, Álvarez Lemos FL, Arriagada Ramos FI (2023) Spino-pelvic radiological parameters: comparison of measurements obtained by radiologists using the traditional method versus spine surgeons using a semi-automated software (Surgimap). Acta Radiol Open 12(5):20584601231177404. 10.1177/20584601231177404 37223123 10.1177/20584601231177404 PMC10201147

[7] Lafage R, Ferrero E, Henry JK, Challier V, Diebo B, Liabaud B, Lafage V, Schwab F (2015) Validation of a new computer-assisted tool to measure spino-pelvic parameters. Spine J 15:2493–2502. 10.1016/j.spinee.2015.08.067 26343243 10.1016/j.spinee.2015.08.067

[8] Ailon T, Scheer JK, Lafage V, Schwab FJ, Klineberg E, Sciubba DM, Protopsaltis TS, Zebala L, Hostin R, Obeid I, Koski T (2016) Adult spinal deformity surgeons are unable to accurately predict postoperative spinal alignment using clinical judgment alone. Spine Deform 4:323–329. 10.1016/J.JSPD.2016.02.003 27927523 10.1016/j.jspd.2016.02.003

[9] Faro FD, Marks MC, Pawelek J, Newton PO (2004) Evaluation of a functional position for lateral radiograph acquisition in adolescent idiopathic scoliosis. Spine 29:2284–2289. 10.1097/01.BRS.0000142224.46796.A7 15480143 10.1097/01.brs.0000142224.46796.a7

[10] Kim H, Kim HS, Moon ES, Yoon CS, Chung TS, Song HT, Suh JS, Lee YH, Kim S (2010) Scoliosis imaging: what radiologists should know. Radiographics 30:1823–1842. 10.1148/rg.307105061 21057122 10.1148/rg.307105061

[11] Lenke LG, Edwards CC, Bridwell KH (2003) The lenke classification of adolescent idiopathic scoliosis: how it organizes curve patterns as a template to perform selective fusions of the spine. Spine 28:S199–S207. 10.1097/01.BRS.0000092216.16155.33 14560193 10.1097/01.BRS.0000092216.16155.33

[12] Doğan NÖ (2018) Bland-altman analysis: a paradigm to understand correlation and agreement. Turk J Emerg Med 18:139–141. 10.1016/J.TJEM.2018.09.001 30533555 10.1016/j.tjem.2018.09.001 PMC6261099

[13] Hardesty CK, Aronson J, Aronson EA, Ranade AS, McCracken CW, Nick TG, Cordell CL (2013) Interobserver variability using a commercially available system of archived digital radiography with integrated computer-assisted measurements for scoliosis Cobb angles. J Pediatr Orthop 33:163–169. 10.1097/BPO.0b013e3182770bd3 23389571 10.1097/BPO.0b013e3182770bd3

[14] Maillot C, Ferrero E, Fort D, Heyberger C, Le Huec JC (2015) Reproducibility and repeatability of a new computerized software for sagittal spinopelvic and scoliosis curvature radiologic measurements: Keops(®). Eur Spine J 24:1574–1581. 10.1007/s00586-015-3817-1 25724685 10.1007/s00586-015-3817-1

[15] Beekman CE, Hall V (1979) Variability of scoliosis measurement from spinal roentgenograms. Phys Ther 59:764–765. 10.1093/PTJ/59.6.764 441122 10.1093/ptj/59.6.764

[16] Sullivan TB, Reighard FG, Osborn EJ, Parvaresh KC, Newton PO (2017) Thoracic idiopathic scoliosis severity is highly correlated with 3D measures of thoracic kyphosis. J Bone Joint Surg Am 99:e54. 10.2106/JBJS.16.01324 28590384 10.2106/JBJS.16.01324

[17] Dang NR, Moreau MJ, Hill DL, Mahood JK, Raso J (2005) Intra-observer reproducibility and interobserver reliability of the radiographic parameters in the spinal deformity study group’s AIS radiographic measurement manual. Spine 30:1064–1069. 10.1097/01.BRS.0000160840.51621.6B 15864160 10.1097/01.brs.0000160840.51621.6b

[18] Mior SA, Kopansky-Giles DR, Crowther ER, Wright JG (1996) A comparison of radiographic and electrogoniometric angles in adolescent idiopathic scoliosis. Spine 21:1549–1555. 10.1097/00007632-199607010-00013 8817783 10.1097/00007632-199607010-00013

[19] Vidal C, Ilharreborde B, Azoulay R, Sebag G, Mazda K (2013) Reliability of cervical lordosis and global sagittal spinal balance measurements in adolescent idiopathic scoliosis. Eur Spine J 22:1362–1367. 10.1007/S00586-013-2752-2 23543370 10.1007/s00586-013-2752-2 PMC3676559

[20] Ilharreborde B, Steffen JS, Nectoux E, Vital JM, Mazda K, Skalli W, Obeid I (2011) Angle measurement reproducibility using EOS three-dimensional reconstructions in adolescent idiopathic scoliosis treated by posterior instrumentation. Spine 36:E1306–E1313. 10.1097/BRS.0b013e3182293548 21697768 10.1097/BRS.0b013e3182293548

[21] Vrtovec T, Ibragimov B (2022) Spinopelvic measurements of sagittal balance with deep learning: systematic review and critical evaluation. Eur Spine J 31:2031–2045. 10.1007/S00586-022-07155-5 35278146 10.1007/s00586-022-07155-5

[22] Löchel J, Putzier M, Dreischarf M et al (2024) Deep learning algorithm for fully automated measurement of sagittal balance in adult spinal deformity. Eur Spine J 33:4119–4124. 10.1007/s00586-023-08109-1 38231388 10.1007/s00586-023-08109-1

[23] Orosz LD, Bhatt FR, Jazini E, Dreischarf M, Grover P, Grigorian J, Roy R, Schuler TC, Good CR, Haines CM (2022) Novel artificial intelligence algorithm: an accurate and independent measure of spinopelvic parameters. J Neurosurg Spine 37:893–901. 10.3171/2022.5.SPINE22109 35901700 10.3171/2022.5.SPINE22109

[24] Korez R, Putzier M, Vrtovec T (2020) A deep learning tool for fully automated measurements of sagittal spinopelvic balance from X-ray images: performance evaluation. Eur Spine J 29:2295–2305. 10.1007/s00586-020-06406-7 32279117 10.1007/s00586-020-06406-7

[25] Yeh YC, Weng CH, Huang YJ, Fu CJ, Tsai TT, Yeh CY (2021) Deep learning approach for automatic landmark detection and alignment analysis in whole-spine lateral radiographs. Sci Rep 11:7618. 10.1038/s41598-021-87141-x 33828159 10.1038/s41598-021-87141-x PMC8027006

